# Current directions in views on ageing

**DOI:** 10.1007/s10433-020-00585-4

**Published:** 2020-10-26

**Authors:** Verena Klusmann, Anna E. Kornadt

**Affiliations:** 1 Department of Psychology, Psychological Assessment and Health Psychology, University of Konstanz, Constance, Germany; 2 Department of Psychology and Human Movement Science, University of Hamburg, Hamburg, Germany; 3 Department of Behavioural and Cognitive Sciences, Université du Luxembourg, Esch-sur-Alzette, Luxembourg

**Keywords:** Age stereotypes, Lifespan development, Self-perceptions of ageing, Subjective ageing, Views on ageing

In the year 2020, the COVID-19 pandemic has posed an unprecedented, worldwide challenge, pairing a dangerous, viral infection with extensive measures designed to contain it. This has led to severely restricted behavioural freedoms and has been accompanied by fears and worries. Particularly striking for us as researchers in the field of views on ageing were the discussions and regulations to contain the pandemic in order to protect older people. Buttressed by our own research, for years we have been committed to the promotion of differentiated views on old age that more accurately represent the reality of ageing and older people: that is, as diverse and multidimensional (Klusmann et al. 2020; Kornadt et al., 2019). In the societal but also in the scientific dialogue surrounding the pandemic, however, older people have been treated as one, barely differentiated risk group; as vulnerable, helpless, and in need of society’s protection. Strict contact restrictions have been imposed on institutions for older people, social structures have been locked down, families have been urged to reduce contact with older relatives, and people over 65 have been temporarily banned from their workplaces and from attending events, both in public and private spaces. Family members have often taken over the daily tasks such as shopping for older relatives, sometimes even without their consent. These events have had and will continue to have a number of psychosocial consequences—as ageing researchers have pointed out on various occasions (e.g. Ayalon et al. 2020; Ehni and Wahl 2020).

Our special section of the European Journal of Ageing (EJA) is devoted to views on ageing, that is, people’s conceptions about ageing and older people that manifest both on the individual as well as on the societal level and that reflect in affect, cognition, and behaviour. All of the articles were written and accepted for publication before the onset of the COVID-19 crisis. The pandemic has, however, certainly amplified the timeliness of our work. Views on ageing not only determine the age-friendliness of people’s thoughts and behaviour, but also their own personality development, preventive action, and health behaviour, not only in old age, but from early life on (Klusmann et al. 2020; Kornadt et al. 2019). The pandemic has poignantly demonstrated the need to advance our understanding of the structure, origins, and consequences of views on ageing in order to enhance well-being across the entire life span.

The special section of this EJA issue is an output of our scientific network *Images of Ageing* (German ‘Altersbilder’), funded by the Deutsche Forschungsgemeinschaft (German Research Foundation, DFG) since 2017. In a number of workshops held across the past several years, we aimed to advance research on views on ageing by establishing a common language, identifying and tackling open questions, exchanging our views with international experts, and sharing ongoing research and preliminary findings. We are now proud to present eight contributions that address important and thus far unresolved issues concerning views on ageing across the life span.

Given the absence of integrative theoretical frameworks that cover different age groups and life phases, Kornadt et al. (2019) present a lifespan approach to views on ageing. Bringing together fundamental principles of lifespan development, existing theories of views on ageing, and a breadth of empirical evidence, we describe how views on ageing are both products and drivers of human development across the entire life span. We elaborate on three specific propositions regarding the development, impact, and the central characteristics of views on ageing during different phases of life, paving the way for future research. In a second paper, Klusmann et al. (2020) present a review and systematisation of 89 different self-report instruments which have been used to measure views on ageing. By describing the measures’ quality and purpose and grouping them into four clusters, the review provides an instructive overview of the available measures and the specific research questions they can answer. The review clearly illustrates that most existing measures are explicit as opposed to implicit and regard age as a status as opposed to ageing as a process. Likewise, views on ageing are mostly conceptualised as a trait as opposed to a malleable state. The majority of measures entail cognitive assessments of views on ageing; manifestations on the behavioural and affective level are less considered. The set of available instruments reflects in a more balanced way; however, that age and ageing are multidimensional and multidirectional, and that views could refer to people’s own and/or to other people’s age and ageing.

Spuling et al. (2019) paper goes on to demonstrate the validity of the views on ageing construct as a whole as well as the differential construct validity of the most commonly used views on ageing measures. Using data from the German Ageing Survey, the authors demonstrate that views on ageing are indeed a unique construct, and not just an expression of people’s physical status or mood, or their general self-efficacy, or their optimism. The authors additionally show that general and domain-specific, multidimensional measures of views on ageing are related but certainly not interchangeable. Bowen et al. (2019) shed light on how views on ageing and self-views may vary across the adult life span. Using data from the Midlife in the US study, the authors compare common age stereotypes with how young, middle-aged, and older adults see themselves and their own age group. Despite confirming the existence of common age stereotypes, the authors find that adults’ self-evaluations are highly positive and largely consistent across age groups. Their results hence demonstrate the stability of the ageing self. Their results also highlight that the “better than average effect”—whereby people tend to perceive themselves more positively than their peers—may depend on age and on whether the considered characteristic represents a relative strength or weakness of the age group.

Two further articles explore views on ageing as drivers of behaviour and development: Based on an online survey with US adults aged 55–87 years, Montepare (2019) finds that identification with one’s age predicts engagement in a number of everyday behaviours (e.g. personal and grooming activities). Age awareness predicts medical activities, such as getting check-ups and screenings, whereas chronological age is generally less predictive of behaviour, but—given governmental regulation of retirement—is indeed related to retirement activities. Using data of the Rutgers Aging and Health longitudinal study, Benyamini and Burns (2019) investigate in how far different views on ageing and health perception measures are associated with one another and with longevity across a 10-year period. They show that age-group identity and perceived nearness to death predict longevity over and above health perceptions, though the unique effects of views on ageing were weaker.

The final two articles of our special section examine views on ageing as products of behaviour and development, specifically in the health domain. Based on data from a combined total of 10,000 participants, Stephan et al. (2019) demonstrate that engaging in more physical activity is associated with feeling younger in the subsequent 8–20 years. The relationship between physical activity and feeling younger appears to be partially mediated by openness to experience and self-rated health. Their findings are in line with the results of a 6-month intervention study which found that physical activity participation prevented increases in ageing dissatisfaction, which can be explained by motivational benefits or mastery experiences (Klusmann et al. 2012). Wurm et al. (2019) investigate how views on ageing may change as a result of a critical health event. In a matched-participants design, they compare about 200 people of the German Ageing Survey who experienced a cardiovascular event to 200 people who did not. The authors find that having a cardiovascular event causes people to view their own ageing more negatively and feel older. The studies of Stephan et al. (2019) and Wurm et al. (2019) demonstrate that views on ageing not only drive health behaviour changes, health, and even mortality as shown by previous studies (e.g. Klusmann et al. 2019; Stephan et al. 2015, 2018; Westerhof and Wurm 2018; Wurm et al. 2010), but, vice versa, are also driven by changes in health.

Together, the articles in our special section of this EJA issue significantly advance our understanding of views on ageing. The special section makes it clear that views on ageing are relevant across the whole life span, and not just in the second half of life. The articles clearly demonstrate the validity of the views on ageing construct and systematise the similarities and distinctions between different views on ageing measures. The articles also fill in some important research gaps regarding the causes and consequences of views on ageing and provide insights into the underlying mechanisms. We hope that our work will encourage more transparent communication within and outside of the field, as well as encourage new and innovative views on ageing research which considers the entire life span.

On that note, our network has identified a number of specific ways views on ageing research could be advanced even further. To study views on ageing with a truly lifespan approach, researchers need valid instruments suitable for measuring views on ageing across different age and target groups that also take into account the fact that views on ageing are multidimensional, domain specific, and multidirectional. This may entail developing not only cognitive measures, but also a better targeting of the affective, behavioural, and physiological levels. We need to know much more about how views on ageing, their reference points, their causes, and their consequences develop and change across the entire life span. We also need more systematic and longitudinal evidence on the dynamic and bidirectional relationships between views on ageing and development across the entire life span in different domains of life.

Finally, coming back to the starting point of our editorial, a central question is what will be the short- and long-term consequences of “COVID ageism” (Kessler and Bowen 2020). By now, many studies have demonstrated that deficit-oriented representations of ageing and older people, even cursory presentations of older people’s deficits in experimental settings, cause older people to perform and feel worse about themselves across a diversity of measures including memory, driving confidence, or walking speed (Chapman et al. 2014; Hagood and Gruenewald 2018; Robertson et al. 2015). Will people internalise the image of older people as vulnerable and in need of protection? What will be the consequences of identifying (or not) with the “at-risk” group of older people during the pandemic? How can we prevent deficit-oriented views on ageing from becoming self-fulfilling prophecies? In particular, in light of the events of this past year, we need to know much more about the mechanisms by which views on ageing affect and are affected by our changing psychosocial contexts, as well as what kind of interventions work for whom and for how long.

Aptly enough, the World Health Organisation declared 2020 the onset of the Decade of Healthy Ageing, which aims to reduce social inequalities related to age (cf. this year’s first EJA editorial by Kliegel et al. 2020). We believe that views on ageing research can contribute to this ambitious and important goal. We thank the editors for the opportunity to realise this special section and for their support and encouragement throughout the process. We hope that you, the readers of the EJA, will enjoy reading our articles and find them as exciting as we do.
